# Reply to “Comment on Comprehensive Nutritional and Dietary Intervention for Autism Spectrum Disorder—A Randomized, Controlled 12-Month Trial, Nutrients 2018, 10, 369”

**DOI:** 10.3390/nu11051138

**Published:** 2019-05-22

**Authors:** James B. Adams, Tapan Audhya, Elizabeth Geis, Eva Gehn, Valeria Fimbres, Elena L. Pollard, Jessica Mitchell, Julie Ingram, Robert Hellmers, Dana Laake, Julie S. Matthews, Kefeng Li, Jane C. Naviaux, Robert K. Naviaux, Rebecca L. Adams, Devon M. Coleman, David W. Quig

**Affiliations:** 1 School for Engineering of Matter, Transport & Energy, Arizona State University, Tempe, AZ 85287, USA; autismstudynurseasu@gmail.com (E.G.); ecgehn@gmail.com (E.G.); Valeria.Fimbres@asu.edu (V.F.); epollard1025@gmail.com (E.L.P.); julieaingram@yahoo.com (J.I.); thebeckyadams@gmail.com (R.L.A.); devon.coleman@asu.edu (D.M.C.); 2 Health Diagnostics, South Amboy, NJ 08879, USA; audhyatk@optonline.net; 3 Southwest College of Naturopathic Medicine, Tempe, AZ 85282, USA; J.Mitchell@scnm.edu; 4 Arizona Allergy Associates, Phoenix, AZ 85004, USA; rhellmers@aol.com; 5 Dana Laake Nutrition, Kensington, MD 20895, USA; danalaake@aol.com; 6 Nourishing Hope, San Francisco, CA 94117, USA; julie@NourishingHope.com; 7 The Mitochondrial and Metabolic Disease Center, University of California, San Diego, CA 92093, USA; kli@ucsd.edu (K.L.); jnaviaux@ucsd.edu (J.C.N.); naviaux@ucsd.edu (R.K.N.); 8 Doctor’s Data, St. Charles, IL 60174, USA; dquig@DoctorsData.com

We thank Vorland et al. [1] for their interest in our research and their critique of our paper [2]. Here are our responses to the specific points that they raised.

We confirm that we previously responded to their inquiries about our study.

First (regarding randomization): We confirm that the participants were enrolled on a rolling basis, matched with participants of similar ages and a similar level of behavioral/Applied Behavioral Analysis (ABA) therapy, and then randomly assigned to one of the two groups. ABA is an important therapy, and we hypothesized that it might affect outcomes in our 12-month study, which is why we attempted to include it as part of the randomization procedure.

Second (regarding siblings): We confirm that siblings were randomized together. The reason is that this was a single-blind study, so the participants knew if they were taking supplements or improving their diet. We felt that it would place an undue burden on the family to provide a different diet and supplements to each sibling, compromising study compliance.

Third (regarding block randomization): There were three sibling pairs who completed the treatment arm, and two sibling pairs who completed the non-treatment arm, out of a total of 28 participants in the treatment arm and 27 participants in the non-treatment arm. Thus, only 21% and 15% of the participants were siblings, respectively. 

We investigated whether removing all siblings affected our results, focusing on the Childhood Autism Rating Scale (CARS) test as a typical and important test. We used the Anderson–Darling test to determine if the treatment groups (with or without siblings) and the non-treatment group (with or without siblings) involved normal distributions, and confirmed that all were drawn from normal distributions (see Table 1). Next, we used the F test to determine if the samples with siblings had equal variance (they did) and if the samples without siblings had equal variance (they did). This means that the assumptions required to apply a t-test were met and the analysis was reasonable for the study design.

Next, we used a one-sided t-test to determine if the treatment group improved more than the non-treatment group. The p-value for the comparison with siblings was 0.033, and the p-value for the comparison without siblings was 0.025. So, the treatment group improved more than the non-treatment group, regardless of whether or not siblings were included.

Registration on clinicaltrials.gov: there was an unfortunate delay in completing the submission to clinicaltrials.gov, and the dates are stated in the original paper.

One-tailed and two-tailed tests: For behavioral tests, our hypothesis was that there was an improvement in ASD-related symptoms, so a one-tailed test was appropriate. For laboratory tests, we instead used two-tailed tests because we did not always have a hypothesis as to which way biomarkers would change.

Additional analyses: Vorland et al. suggest some interesting additional analyses and sub-analyses that could be conducted, but our sample size is too small, so those analyses would be under-powered. Those suggestions can be considered in future studies.

In closing, we thank Vorland et al. for their comments and the opportunity to provide more detail on our study.

## Figures and Tables

**Table 1:** Summary of Anderson–Darling test for normality, F test for equal variance, and t-test for equal variance.

**Siblings Included**	**Anderson–Darling Test for Normality**	**Equal Variance (F test)**
Treatment	p=0.337 (accept)	p=0.095 (accept)
Non-Treatment	p=0.824 (accept)	
$H_{0}:\mu_{1}=\mu_{2}$$H_{1}:\mu_{1}>\mu_{2}$ t-test for equal variance: p=0.033 (accept)		
**Siblings Excluded**	**Anderson-Darling Test for Normality**	**Equal Variance (F test)**
Treatment	p=0.582 (accept)	p=0.096 (accept)
Non-treatment	p=0.894 (accept)	
$H_{0}:\mu_{1}=\mu_{2}$$H_{1}:\mu_{1}>\mu_{2}$ t-test for equal variance: p=0.025 (accept)		
